# Does oval oocyte have an impact on embryo development in in vitro fertilization?

**DOI:** 10.5935/1518-0557.20170005

**Published:** 2017

**Authors:** Binarwan Halim, Hilma Putri Lubis, Diana Novia, Masithah Thaharuddin

**Affiliations:** 1 HFC IVF center Division of Reproductive, Endocrinology and Infertility Department of Obstetrics and Gynecology, Faculty of Medicine University of Sumatera Utara, Medan, Indonesia

**Keywords:** *In vitro* fertilization, normal oocyte, oval oocyte, dimorphism, morphology

## Abstract

**Objective:**

To compare the outcomes of embryo development between oval-shaped oocyte as an abnormal morphology oocyte and a normal morphology oocyte in In Vitro Fertilization (IVF).

**Methods:**

This study was a comparative analytical study with retrospective approach which lasted from September 2014 until November 2015. For this study, we used secondary data (medical records) from 24 patients submitted to IVF at the Halim Fertility Center. The oocyte morphology was divided into two groups: normal-shaped oocyte and oval-shaped oocyte.

**Results:**

Our study included 120 oocytes with 60 oval-shaped oocytes and 60 normal oocytes. We found fertilization rates of 68.3% in the normal oocyte group and 61.7% in the oval-shaped oocyte group; and there was no significant difference between the normal oocyte group and the oval-shaped oocyte group (*p* > 0.05). In the normal oocyte group, 65% had reached day 3 embryos, and in the oval-shaped oocyte group it was 50%, with no significant difference between the groups (*p* > 0.05). We also found 46.7% transferrable embryos in the oval oocytes group compared to 63.3% in the normal oocytes group, with no significant difference between the groups (*p* > 0.05).

**Conclusion:**

There was no significant difference in fertilization rates and embryo quality between normal morphology oocytes and oval-shaped oocytes.

## INTRODUCTION

Human reproduction is the result of a union between two highly specialized cells- the oocyte and the spermatozoon. Of these, the oocyte deserves special mention because of its key functions; it receives the spermatozoon during fertilization, contributes much of the cytoplasm for early embryo development and provides half of the genome for one the resulting zygotes (Savitha *et al*., 2012; Plachot, 2000).

The correlation between oocyte quality, embryo development, and *in-vitro* fertilization (IVF) outcomes has been widely studied (Lazzaroni-Tealdi *et al*., 2015). Some studies revealed that successful pregnancy outcome with IVF/intracytoplasmic sperm injection (ICSI) depended on several variables including oocyte and sperm quality (Savitha *et al*., 2012; Braga *et al*., 2013; Ebner *et al*., 2006).

Oocyte quality has been regarded as a variable that may influence embryo quality (Plachot, 2000). Oocyte and embryo quality were assessed based on morphological features (Lazzaroni-Tealdi *et al*., 2015; Braga *et al*., 2013; ALPHA Scientists In Reproductive Medicine & ESHRE Special Interest Group Embryology, 2011). However, the correlation between these ''abnormal'' oocyte morphotypes, fertilization rates, and embryo development is still unclear. The most frequently observed oocyte morphologic variations are cytoplasmic changes, including granularity, darkness, vacuole presence. Extracytoplasmic variations are oocyte shape, zona pellucida (ZP), perivitelline space (PVS), and polar body morphology (Savitha *et al*., 2012; Lazzaroni-Tealdi *et al*., 2015; Braga *et al*., 2013). Oocyte dimorphism such as oval (ovoid) oocyte or other abnormal oocyte morphology may affect fertilization rate and embryo development (Ebner *et al*., 2000; 2008; Xia, 1997; Serhal *et al*., 1997). Morphological Oocyte abnormalities also correlated to aneuploidy embryo in IVF cycle (Dolgushina *et al*., 2015). The aim of this study was to compare outcomes of embryo development between oval-shaped oocytes and normal morphology oocytes in IVF.

## MATERIAL AND METHODS

This study was a comparative analytical study with a retrospective approach, which ran from September 2014 until November 2015. The subject of the study was secondary data (medical records) recruited from 24 patients who underwent IVF procedure at the Halim Fertility Center, Division of Reproductive Endocrinology and Infertility, Department of Obstetrics & Gynecology, Faculty of Medicine - University of Sumatera Utara, Haji Adam Malik General Hospital, Medan, Indonesia. Oocyte and embryo quality were assessed based on morphological features. Oocyte morphology was divided into two groups: normal morphology oocyte and oval-shaped oocyte. Samples were selected consecutively to reach the minimum samples requirement based on the inclusion and exclusion criteria. The Inclusion criteria were all woman who underwent IVF procedure with PCOS, tubal factor, endometriosis, idiopathic infertility and male factor. The exclusion criterion was incomplete medical records.

This study was approved by the ethics committee of the faculty of Medicine of the University of Sumatera Utara.

### Controlled Ovarian Hyperstimulation

The standard protocol for controlled ovarian stimulation was the short protocol, carried out using recombinant follicle-stimulating hormone (FSH) (Gonal-F; Serono), 150-300 IU as a daily dose, starting on day 2 of the cycle. We used a gonadotropin-releasing hormone (GnRH) antagonist (Cetrotide; Serono), at a dose of 0,25 mg to prevent a premature luteinizing hormone (LH) surge, starting when at least one follicle > 14 mm was visualized. Follicular growth was monitored using transvaginal ultrasound, starting on day 4 of the gonadotropin administration. The average of stimulation duration was 10-12 days. Oocyte maturation was triggered with recombinant hCG (Ovidrel; Serono), when the lead follicles reached 17-18 mm. Oocytes were retrieved 36 hours after the injection of hCG by transvaginal ultrasound guiding the needle aspiration of follicles.

### Oocyte Morphology Assessment

Retrieved oocytes were maintained in culture medium supplemented (G-IVF^TM^ Plus, Vitrolife) with 10% protein supplement and covered with paraffin oil for 2 to 3 hours before removing cumulus cells. The surrounding cumulus cells were removed after exposure to an N-2-hydroxyethylpiperazine-N0-2-ethanesulfonic acid (HEPES)-buffered medium containing hyaluronidase (SynVitro^TM^ Hyadase, Origio). The remaining cumulus cells were mechanically removed by gently pipetting with a hand-drawn Pasteur pipette. Oocyte morphology was assessed just before sperm injection (4 hours after retrieval) using an inverted Nikon Diaphot (stereomicroscope Nikon smz 1000+) microscope. The oocytes were classified as normal and abnormal (oval) according to their morphologic appearance.

### ICSI Procedure

The Intracytoplasmic Sperm Injection was performed in a microinjection dish prepared with 10-µL droplets of buffered medium (G-MOPS Plus, Vitrolife) and covered with paraffin oil on the heated stage of an inverted microscope (37 ± 0.5ºC) (Nikon Eclipse TE2000). Approximately 17 hours after ICSI, fertilization was confirmed by the presence of two pronuclei and the extrusion of the second PB. The embryo was cultured at 37 ºC with the level of CO_2_ at 6% and O_2_ at 5%. We used ISM1 medium (MediCult) and cultured until day 3 (approximately 68 hours), then we performed fresh or freezing embryo transfer.

### Embryo Morphology Assessment

Embryo morphology was assessed at the zygote stage (16–18 hours after ICSI) and on the mornings of days 2 and 3 of embryo development, using an inverted Nikon Diaphot microscope (stereomicroscope Nikon smz 1000+) with a Hoffmann modulation contrast system under x200 magnification. Immediately before ET we assessed the embryo morphology.

To evaluate the cleavage-stage morphology, the following parameters were recorded based on the Istanbul consensus workshop on embryo assessment (ALPHA Scientists In Reproductive Medicine & ESHRE Special Interest Group Embryology, 2011): the number of blastomeres, the percentage of fragmentation, the variation in blastomere symmetry, the presence of multinucleation, and defects in the ZP and cytoplasm. The high-quality cleavage stage embryos were defined as those with all the following characteristics: 4 cells on day 2 or 8–10 cells on day 3, < 10% fragmentation, symmetric blastomeres, the absence of multinucleation, colorless cytoplasm with moderate granulation and no inclusions, the absence of PVS granularity, and the absence of ZP dimorphism. Embryos lacking any of these characteristics were of low quality (Table 1).

**Table 1:** Consensus scoring system for cleavage- stage embryos (in addition to cell number) (ALPHA Scientists In Reproductive Medicine & ESHRE Special Interest Group Embryology, 2011)

Grade	Rating	Description
1	Good	< 10% fragmentation
		Stage-specific cell size
		No multinucleation
2	Fair	10 - 25% fragmentation
		Stage-specific cell size for majority of cells
		No evidence of multinucleation
3	Poor	Severe fragmentation (.25%)
		Cell size not stage specific
		Evidence of multinucleation

### Data Analysis

We tabulated data on oocyte morphology and embryo development. Embryo development outcomes were determined by fertilization rates, cleavage rates on day 3 and transferrable embryo. Data was analyzed by computer applications in accordance with comparative analytical data presentations. To analyze embryo development outcomes between normal morphology oocyte and oval morphology oocyte, we used the chi-square statistical test with a 95% confidence interval. A P value of < 0.05 was considered statistically significant.

### Sample size Calculation

In this study, we used a comparative analytical study with retrospective approach. Therefore, this was the sample size calculation.

$$n_{1}=n_{2}=\frac{\left(Z\alpha \sqrt{2\mathrm{PQ}}+Z\beta {\sqrt{P_{1}Q_{1}+P_{2}Q_{2}}}^{2}\right)}{{\left(P_{1}-P\right)}_{2}^{2}}$$

Zα = normal default value of Z table that depends on the value of α is determined. α value = 0.05 → Zα = 1.96

Zβ. = normal default value of table Z that depends on the value of β is determined. β value = 0,20 → Zβ = 0.84

P1 = the proportion of fertilized oocytes with normal morphology= 0.9

P2 = the proportion of fertilized oocytes with oval shape = 0.6

Q1 = 1- P1 = 0.1

Q = 1- P = 0.2

Q2 = 1- P2 = 0.3

P = (P1+ P2)/2 = 0.8

n1 = n2 = total sample = 32 oocytes (minimum total sample)

Therefore, the total sample in this study was 60 oocytes (each sample)

## RESULTS

From September 2014 until November 2015, we included 120 oocytes recruited from 24 patients, being 60 with oval shape and 60 normal morphology oocytes.

From table 2, the fertilization rate in the normal morphology oocyte group was 68,3% in 41 oocytes and the fertilization rate in the oval-shaped oocyte group was 61.7% in 37 oocytes. The Statistical analysis showed no significant difference in fertilization rates between the normal oocyte group and the oval oocyte group (*p* = 0.444).

**Table 2:** Fertilization rate , Cleavage outcome at day 3 and Embryo quality between normal oocyte and oval oocyte

Oocyte morphology	Fertilization rate		*p* value	Cleavage		*p* value	Embryo quality		*p* value
	Positive	Negative		Positive	Negative		Untransferrable embryo	Transferrable embryo	
Normal	41 (68.3%)	19 (31.7%)	0.444*	39 (65%)	21 (35%)	0.097*	22 (36.7%)	38 (63.3%)	0,067*
Oval	37 (61.7%)	23 (38.3%)		30 (50%)	30 (50%)		32 (53.3%)	28 (46.7%)	
Total	78 (65%)	42 (35%)		69 (57.5%)	51 (42.5%)		54 (45%)	66 (55%)	

* chi-square

This table also showed that the cleavage outcome at day 3 in the normal oocyte group was 65% in 39 oocytes and in the oval-shaped oocyte group it was 50% in 30 oocytes, with no significant difference between the groups (*p* = 0.097).

The transferrable embryo ratio from the normal morphology oocyte group was 63.3% and the transferrable embryo in the oval-shaped oocyte group was 46.7%. The Statistical analysis showed no significant difference in embryo quality between the normal oocyte group and the oval-shaped oocyte group (*p* = 0.067).

## DISCUSSION

Oocyte quality is an important prognostic factor, since the nuclear and cytoplasmic maturity of the oocyte may be directly related to the success rate of ICSI (Ebner *et al*., 2006; Kahraman *et al*., 2000). Oocyte and embryo quality were assessed based on morphological features. a dysmorphic oocyte can be regarded as a lower quality oocyte. Dysmorphic oocytes were classified as having a dark cytoplasm, cytoplasmic granularity, cytoplasmic vacuoles, refractive bodies in the cytoplasm, smooth endoplasmic reticulum in the cytoplasm, an oval shape, an abnormal zona pellucida, a large perivitelline space, debris in the perivitelline space, or an abnormal polar body (PB). An oval-shaped oocyte is a dysmorphic oocyte in the IVF cycle. Two possible mechanisms may account for the occurrence of ovoid oocytes: mechanical stress during oocyte puncture and/or the denudation process that deforms the oocyte (Ebner *et al*., 2008).

Our study reveals that oval oocytes, when compared to normal oocytes, do not yield any difference in fertilization rates, cleavage rates on day 3 and embryo quality. De Sutter *et al*. (1996) suggested that the morphology of the oocyte bears no relationship to fertilization after ICSI. Balaban *et al*. (1998) also reported that abnormal oocyte morphology is not associated with a decreased fertilization rate or unfavorable embryo quality. They also stated that embryos derived from abnormal oocytes yield similar clinical pregnancy and implantation rates when transferred, compared with embryos derived from normal oocytes.

Although poor quality oocytes are difficult to fertilize, there is no correlation between ICSI fertilization success and oocyte dimorphism. A failed fertilization in ICSI may occur either because of oocyte activation failure or the oocytes were activated but failed to complete the events leading to fertilization. Another possibility is the unfertilized oocyte containing a decondensed sperm head, indicating failure of oocyte activation, and not a technical error. Savitha *et al*. (2012) reported that oocyte abnormality can be directly influenced by underlying infertility, effects of ovarian hyperstimulation, and advanced maternal age.

Xia (1997) reported that human oocyte grading can be based on three factors, which are: polar body, size of perivitelline space and cytoplasmic inclusions, and these were significantly related to fertilization rate and embryo quality after ICSI. Loutradis *et al*. (1999) found that oocyte morphology correlates well with embryo quality and pregnancy rates after ICSI.

Serhal *et al*. (1997) reported that the outcome of ICSI was dependent on the quality of the oocytes retrieved, they also stated that normal fertilization and early embryo development were achieved in oocytes with abnormal cytoplasm morphology, but the embryos failed to demonstrate the same implantation potential as those derived from oocytes with normal cytoplasm.

Meriano *et al*. (2001) reported that oocyte dimorphisms were not associated with adverse ICSI outcomes. Khalili *et al*. (2005) stated that oocyte quality plays a major role in the fertilization process and embryo development in ART programs. Rienzi *et al*. (2008) reported that morphologic evaluation before ICSI helps to identify MII oocytes with higher developmental potential.

Ebner *et al*. (2008) stated that Ovoid oocytes with abnormal cleavage pattern show delayed preimplantation development probably due to a reduced number of cell-to-cell contacts. Setti *et al*. (2011) reported that the presence of large polar bodies, large PVS, refractive bodies or vacuoles are associated with decreased oocyte fertilization. They found that the effects of oocyte abnormalities on implantation and pregnancy rates remain unclear.

Braga *et al*. (2013) stated the individual identification of oocyte dimorphisms might be a prognostic tool for blastocyst development and quality. Yu *et al*. (2015) reported that the fertilization rate was significantly lower in dysmorphic oocytes than in normal-shaped oocytes, but embryo quality in dysmorphic oocyte group and normal form oocyte group at day 3 was similar.

Fertilization rates, cleavage rates and high quality embryos were not affected by abnormal oocyte morphology but implantation rate was found to be higher in normal morphology oocytes than abnormal morphology oocytes. But we still think that evaluation of MII oocyte quality before ICSI is recommended, leading to higher embryo development.

## CONCLUSION

There is no significant difference in fertilization rates and embryo quality between normal morphology oocytes and oval-shaped oocytes.
